# Comparison of Antioxidant Properties of Refined and Whole Wheat Flour and Bread 

**DOI:** 10.3390/antiox2040370

**Published:** 2013-11-25

**Authors:** Lilei Yu, Anne-Laure Nanguet, Trust Beta

**Affiliations:** 1 Department of Food Science, University of Manitoba, Winnipeg, MB R3T 2N2, Canada; E-Mails: umyul@cc.umanitoba.ca (L.Y.); annelaure.nanguet@yahoo.fr (A.-L.N.); 2 Higher Engineering School in Agri-Food Integrated Development (ESIROI), Sainte-Clotilde, Reunion Island 97490, France; 3 Richardson Centre for Functional Foods & Nutraceuticals, Smartpark, University of Manitoba, Winnipeg, MB R3T 2N2, Canada

**Keywords:** wheat, whole wheat flour, refined flour, bread, antioxidant activity, ferulic acid

## Abstract

Antioxidant properties of refined and whole wheat flour and their resultant bread were investigated to document the effects of baking. Total phenolic content (TPC), 2,2-diphenyl-1-picrylhydrazyl (DPPH) radical scavenging activity and oxygen radical absorbance capacity (ORAC) were employed to determine the content of ethanol extractable phenolic compounds. HPLC was used to detect the presence of phenolic acids prior to their confirmation using LC-MS/MS. Whole wheat flour showed significantly higher antioxidant activity than refined flour (*p* < 0.05). There was a significant effect of the bread-making process with the TPC of whole wheat bread (1.50–1.65 mg/g) and white bread (0.79–1.03 mg/g) showing a respective reduction of 28% and 33% of the levels found in whole wheat and refined flour. Similarly, baking decreased DPPH radical scavenging capacity by 32% and 30%. ORAC values, however, indicated that baking increased the antioxidant activities of whole wheat and refined flour by 1.8 and 2.9 times, respectively. HPLC analysis showed an increase of 18% to 35% in ferulic acid after baking to obtain whole and refined wheat bread containing 330.1 and 25.3 µg/g (average), respectively. Whole wheat flour and bread were superior to refined flour and bread in *in vitro* antioxidant properties.

## 1. Introduction

Recently, epidemiological studies have shown that the consumption of whole grains and grain-based products is associated with the reduced risk of oxidative-stress related chronic diseases and age-related disorders, such as cardiovascular diseases, carcinogenesis, type II diabetes and obesity [1]. Parts of the health benefits of whole grain flours are attributed to the presence of antioxidants. In addition to the most common antioxidants, such as vitamin C (tocopherols and tocotrienols), vitamin E and carotenoids [2], grains also contain some phyto-antioxidants, including phenolic acids and flavonoids [3]. The most abundant antioxidants in whole grains are phenolic acids, which are highly concentrated in the bran and the germ [2], both of which are removed to obtain refined flour. Phenolic acids exist as free, esterified and insoluble-bound forms [4]. One of the advantages of bound phytochemicals is their ability to survive digestion in the upper gut, allowing them to reach the colon and, therefore, exert health benefits [5]. Phenolic compounds present in cereals are primarily ester-linked to cell wall polymers representing 80%–95% of the total amount [6]. Ferulic acid is one example with free, conjugated and bound forms in the ratio of 0.1:1:100 [5]. The cell wall bound phenolics are alkali labile [7]. Therefore different extraction procedures are needed to obtain free and bound phenolic acids.

Unlike laboratory milled flour, commercially milled flour often contains other additives, for example, ascorbic acid, amylase and azodicarbonamide in whole wheat flour. In comparison, to obtain refined flour, the nutrient-rich components of wheat, namely the bran and germ, are usually removed. These components contain phenolic acids, such as ferulic acid and caffeic acid, as well as some other compounds, including phytic acid, vitamin E and selenium, which all contribute to the antioxidant properties of the flour. To compensate for the loss of nutrients, government regulations dictate the addition of vitamins and minerals into these flours and label the modified flour as refined flour. These additives are primarily niacin, reduced iron, thiamine mononitrate, riboflavin and folic acids [8]. As practiced with whole wheat flour, ascorbic acid, amylase and azodicarbonamide are also added.

Commercial flour as the direct raw material that consumers can get from the marketplace needs further exploration of its potential health benefits in terms of antioxidant properties. Even though additives may exert antioxidant effects, they still differ from natural sources on a chemical and biochemical level. Moreover, the bread making process is believed to compromise the nutrient content, for example, leading to the loss of 30% of vitamin E [9]. The literature also indicates that the non-nutrient substances, phenolic acids, are labile to high temperature [10]. On the contrary, baking has been reported to increase the antioxidant activity of wholemeal bread compared with its flour and that the crust of white bread contained slightly more phenolic compounds than the crumb, because of the Maillard reaction [11]. Therefore, the objective of this study was to investigate the antioxidant properties of whole wheat and refined flours and the effect of the baking process on their resultant breads.

## 2. Experimental Section

### 2.1. Materials and Chemicals

Five brands of flours were selected on the basis that each brand contains whole wheat and refined (enriched, white) flour. These brands were namely Robin Hood (Smucker Foods of Canada Corporation, Markham, ON, Canada), Rogers (Rogers Foods Limited, Armstrong, BC, Canada), No Name (Loblaws Inc., Calgary, AB, Canada), Great Value (Wal-Mart Stores, Inc., San Bruno, CA, USA) and Compliments (Sobeys Inc., Whitby, ON, Canada). 

Phenolic acid standards (gallic, protocatechuic, p-hydroxybenzoic, vanillic, caffeic, syringe, p-coumaric, ferulic, sinapic, iso-ferulic, o-coumaric, trans-cinnamic acid) were used for phenolic acid identiﬁcation, and only ferulic acid was quantified (Sigma-Aldrich Chemical Co., St. Louis, MO, USA). Folin–Ciocalteu reagent, 2,2-diphenyl-1-picrylhydrazyl (DPPH), 2,20-azobis (2-methylpropionamide) dihydrochloride (AAPH) and 6-hydroxy-2,5,7,8-tetramethychroman-2-carboxylic acid (Trolox) were applied for antioxidant potential evaluation (Sigma-Aldrich Chemical Co., St. Louis, MO, USA). Ethanol, acetone and ethyl acetate were used for sample preparation and deionized distilled water, HPLC grade methanol and acetic acid were used in HPLC and HPLC-MS/MS analysis.

### 2.2. Bread-Making Method

Dough was prepared using an optimized straight-dough bread-making method [12]. Initially, 5.3 g yeast was dissolved in water and mixed with dry ingredients, including 6.0 g sucrose, 1.5 g salt and 3.0 g shortening. The latter was first melted prior to mixing with other ingredients. Then, 100 g of flour were added to the mixture contained inside the KitchenAid pin-type mixer (Whirlpool Corporation, Benton Charter Township, MI, USA) at low speed for 2 min, followed by 6 min of high speed mixing. During mixing, water was added, depending on the type of flour, to reach the point of minimum mobility. After adequate mixing of the dough, it was placed in a water bath at a temperature of 30 °C. After 90 min rest, the dough was punched manually and rolled using a rolling pin before incubating for 52 min. A second punch was done followed by 25 min incubation. Afterwards, dough was shaped and incubated for the last 33 ± 2 min of proof to a desired height. The oven was preheated to the set temperature of 215 °C prior to baking the dough for 24 min. Each sample was baked in two batches, and the average value was reported.

### 2.3. Sample Preparation

The bread produced from the above flour materials was freeze-dried and ground. Both raw flours and ground bread were milled to pass through a 0.5 mm sieve. Raw flours were measured on an as is basis, and their moisture contents were used to convert the results to a dry basis.

### 2.4. Extraction of Soluble Phenolic Compounds

The antioxidant components of both raw flours and ground bread samples were extracted following the method according to Li *et al*. [13] with some modifications. One gram finely ground flour was mixed with 10 mL 1 N HCl/95% ethanol (v/v, 15/85) solvent in an amber bottle under nitrogen. The mixture was vortexed. Then, extraction was performed in a temperature-controlled (65 °C) water bath shaker (VWR International, Radnor, PA, USA) at a constant speed for 80 min. The resulting mixture was centrifuged at 7800× *g* (10,000 rpm at 5 °C for 15 min). The supernatant was collected and stored in the dark at −20 °C until their use for determination of total phenolic content (TPC), DPPH radical (DPPH•) scavenging activity and oxygen radical absorbance capacity (ORAC). Extraction was done in duplicate for both raw flours and ground bread samples. For HPLC analysis of acidified ethanol extract, the collected supernatant was further dehydrated with 1 g of Na_2_SO_4_ and then filtered with filter paper (125 mmφ, Whatman™ Cat No. 1004 125). The dehydrated samples were concentrated under vacuum using a rotary evaporator (RE-51 Rotary Evaporator, Yamato Scientific America, Inc., Santa Clara, CA, USA). The dried phenolic extract was redissolved in 2 mL of 50% ethanol and filtered with a 0.45 μm syringe filter before HPLC analysis for phenolic acids. Extraction was done in duplicate for raw flours and ground bread samples.

### 2.5. Total Phenolic Content Determination

Total phenolic content of each extract was determined using the Folin–Ciocalteu method as described by Li *et al*. [13]. Briefly, a 10-fold dilution of Folin–Ciocalteu reagent was prepared just prior to use. Then, 1.5 mL of freshly diluted Folin–Ciocalteu reagent was used to oxidize 0.2 mL sample extracts. After allowing the mixture to equilibrate for 5 min, the reaction was then neutralized with 1.5 mL sodium carbonate solution (60 g/L) at room temperature. The absorbance of the resulting solution was measured at 725 nm after 90 min against a blank of acidified ethanol (1 N HCl/95% ethanol, v/v, 15/85). Ferulic acid was used as a standard. Therefore, the total phenolic content of samples was expressed as milligrams of ferulic acid equivalents/g.

### 2.6. DPPH Radical Scavenging Activity Assay

The DPPH method was used according to the modified method of Beta *et al*. [14]. A 60 μmol/L DPPH• reactant was made in methanol. Then 3.9 mL of DPPH• solution was added to 0.1 mL of sample, and the absorbance at 515 nm was measured at *t* = 60 min. To determine the absorbance at *t* = 0 min, measurement was immediately taken after adding 3.9 mL of DPPH• solution to 0.1 mL methanol. The antioxidant activity was calculated as:

% DPPH • scavenging activity = (1 − [*A*_sample,t_/*A*_control,t_ = _0_]) × 100 (1)

A plot of trolox concentration with % DPPH• scavenging activity was used as the standard curve. Based on this curve, the concentrations of flour samples were expressed as micromole equivalent of trolox/g.

### 2.7. Oxygen Radical Absorbance Capacity (ORAC) Assay

The antioxidant activity was determined using the ORAC assay described by Huang *et al*. [15] with some modification. The assay was performed using an FLx800 microplate fluorescence reader, while the software, KC4 3.0, version 29, was used to control the program. Each well in the plate contained 150 µL fluorescein with 25 µL extracts or trolox standard and was incubated at 37 °C for 15 min. After incubation, 25 µL AAPH was added to each well. The fluorescence intensity was then measured automatically by the reader and a regression equation between the trolox concentration and the net area under the fluorescence decay curve was obtained. Therefore:

AUC = 0.5 + *f*_1_/*f*_0_ +...+ *f*_i_/*f*_0_ +...+ *f*_49_/*f*_0_ +0.5 × (*f*_50_/*f*_0_) (2)

where, *f*_0_ = initial fluorescence reading at 0 min and *f*i = fluorescence reading at time *i* min.

The final results were expressed as trolox equivalent based on the standard curve.

### 2.8. Extraction of Insoluble Phenolic Compounds after Alkaline Hydrolysis

The bound phenolic compounds of both raw flours and ground bread samples were extracted following the method according to Chiremba *et al*. [16] with some modifications. A 200 mg finely ground flour sample was extracted with 5 mL of 4 M NaOH using a 45 mL PTFE acid digestion bomb vessel (Parr Instrument Co., Moline, IL, USA). The bomb vessel was placed in a 1400 W domestic microwave oven (Diplomat model D811, Danby, Suweon, Korea). Samples were digested for 45 s at power 8. The hydrolysate was adjusted to a pH of 1.8–2.0 using 6 N HCl and extracted two times with 25 mL of ethyl acetate for 15 min under magnetic stirring. Lastly, 20 mL of ethyl acetate were used to extract for 10 min. The organic phase (supernatant) was removed with a separating funnel. Further dehydration was performed by adding 1 g of Na_2_SO_4_ and then filtered with a filter paper (125 mmφ, Whatman™ Cat No. 1004 125). The extract was concentrated under vacuum using a rotary evaporator (RE-51 Rotary evaporator, Yamato Scientific America, Inc., Santa Clara, CA, USA). The dried phenolic extract was redissolved in 2 mL of 50% ethanol and filtered with a 0.45 μm syringe filter before HPLC analysis for bound phenolic acids. Extraction was done in duplicate for raw flours and ground bread samples.

### 2.9. High Performance Liquid Chromatography (HPLC) Analysis

HPLC analysis of phenolic acids was performed on a Waters model 600 pump and controller with a Waters 2489 UV/visible detector (Waters Corp., Milford, MA, USA) according to Hirawan *et al*. [17]. A Gemini 5 μ C18 110 A column was used (150 × 4.60 mm) (Phenomenex, Torrance, CA, USA), and the solvents were 1% acetic acid in water (solvent A) and 1% acetic acid in methanol (solvent B). Phenolic acid separation was achieved using a 30-min linear solvent gradient at a flow rate of 1.0 mL/min. The solvent gradient was as follows: at 0 min, 80% A, 20% B; 0–8 min, 78% A, 22% B; 8–10 min, 74% A, 26% B; 10–22 min, 73% A, 27% B; 22–24 min, 80% A, 20% B. The detector was set at 320 nm and 280 nm. The injection volume was 10 µL. Identification of phenolic acids was achieved by comparison of the retention time of the phenolic acid standards and samples spiked with phenolic acid standards. Ferulic acid quantitation was based on the standard curves, and the peak area was used for calculations. The precision, validity and repeatability of the HPLC method were checked on an inter- and intra-day basis. The coefficient of variation was less than 0.05. The limit of detection was 0.1 μg/mL. The concentrations of standards used for the calibration curve were 0.5 μg/mL, 1.0 μg/mL, 5 μg/mL, 10 μg/mL, 50 μg/mL, 100 μg/mL, 150 μg/mL, 200 μg/mL, 250 μg/mL and 500 μg/mL. The HPLC analyses were done in duplicate.

### 2.10. LC-MS/MS

The chromatographic separation was carried out according to Hirawan *et al*. [18] on an HPLC (Waters 2695) system equipped with a photodiode array detector (PDA) (Waters 2695) and autosampler (Waters 717 plus) and coupled to a quadrupole time-of-flight mass spectrometer (Q-TOF MS) (Waters Corp, Milford, MA, USA). The analytical column was a 150 × 4.60 mm, Gemini 5 μ C18 110 Å column (Phenomenex, Torrance, CA, USA). The mobile phase consisted of solvent A (1% acetic acid in water) and solvent B (1% acetic acid in methanol). Prior to introduction into the Q-TOF MS, the same 30 min linear solvent gradient was programmed to elute the sample through the column with a flow rate of 1.0 mL/min. A 10 µL sample solution was loaded and injected by the autosampler. The Q-TOF MS was calibrated with sodium iodide for the negative mode through the mass range of 100–1500. Full mass spectra were recorded in negative mode by using the capillary voltage of 700 V and a cone voltage of 30 V. The flow rates of desolvation gas (N_2_) and cone gas (N_2_) were 900 L/h and 50 L/h, respectively. The desolvation temperature and the source temperature were set at 300 °C and 150 °C, respectively. The MS/MS spectra were acquired by using collision energy of 20 V.

### 2.11. Statistical Analysis

The experimental data were subjected to an analysis of variance using the SAS statistical software, version 9.3 (SAS Institute Inc., Cary, NC, USA). All data were reported as the means ± standard deviation (SD) of duplicate analyses. Scheffe’s test was used to determine the significant differences amongst flour and bread sample means at the level of 0.05 (*p* < 0.05).

## 3. Results and Discussion

### 3.1. Total Phenolic Content of Solvent Extractible Compounds

**Figure 1:**
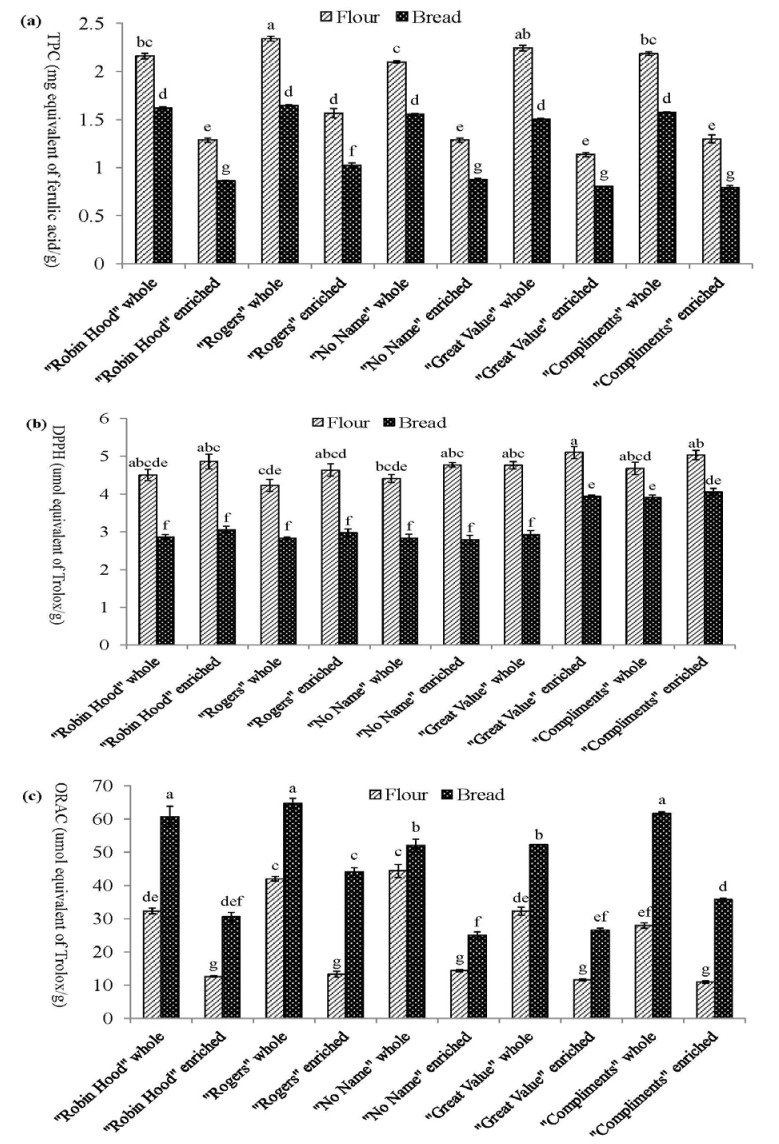
(**a**) Total phenolic content (TPC) (**b**) 2,2-diphenyl-1-picrylhydrazyl (DPPH) radical scavenging activity and (**c**) oxygen radical scavenging absorbance capacity (ORAC) of soluble phenolic compounds from flours and corresponding breads *.

Total phenolic content (TPC) was expressed as milligrams of ferulic acid equivalent (FAE) per gram (mg/g) of dry flour samples (Figure 1a). Ferulic acid, the major phenolic acid found in wheat, was used as a standard. The TPC of refined flours, which ranged from 1.16 to 1.55 mg FAE/g (mean 1.31 mg FAE/g), were significantly lower than those of whole wheat flours (range 2.10 to 2.35 mg FAE/g, mean 2.20 mg FAE/g).

The antioxidant activities of the commercial wheat flours were higher than values reported for the flours milled from soft and hard wheat. The TPC of organic-solvent extractable phenolic compounds was reported to be 0.353 mg FAE/g for hard whole wheat flour and 0.478 mg FAE/g for soft whole wheat flour, while white wheat flours contained 0.137 mg FAE/g and 0.161 mg FAE/g for hard and soft wheat, respectively [19]. The lower values presented could be attributed to the defatting step, which removed some lipophilic phenolic compounds. Liu and others [20] reported the TPC of six different bread wheat (*Triticum aestivum*) grains. The values ranged from 1.46 to 2.26 mg/g, which were similar to our results. Significant differences were detected among different brands, indicating that these wheat flours may exhibit different levels of antioxidant activities.

The average TPC for bread made from whole wheat and refined flour were 1.58 and 0.87 mg FAE/g, respectively. The lower TPCs of 1.01 and 0.52 mg FAE/g have been reported, respectively, for purple wheat bread from whole and refined flour [21]. Overall, the TPC of bread decreased to about 72% and 67% of the average content found in whole wheat and refined flour. This was likely due to the loss of some phenolic acids, which have been reported as labile to baking, and the loss of some reducing vitamins, such as vitamin C (including ascorbic acid, which was added as a mandatory vitamin enrichment) [10,22]. 

* Results are obtained based on acidified aqueous ethanol-soluble extracts and expressed as mean ± standard deviation; Different letters in each bar are significantly different at *p* < 0.05 (Scheffe’s test).

### 3.2. DPPH Radical Scavenging Activity of Soluble Phenolic Compounds

The DPPH radical scavenging activities of flour samples were expressed as micromole trolox equivalents per gram (µmol TE/g) of dried flours. The results are summarized in Figure 1b. Refined flours (range 4.59 to 5.00 µmol TE/g, mean 4.82 µmol TE/g) had slightly higher values than their whole wheat flour counterparts (range 4.28 to 4.77 µmol TE/g, mean 4.53 µmol TE/g), but the differences were not significant. Lavelli *et al*. [23] reported the radical scavenging activities of 63 einkorn wholemeal flours against the DPPH radical using water-saturated l-butanol as the extracting solvent. The values (0.58 to 1.46 µmol TE/g) were significantly lower than our results. On the contrary, Liu and others [20] reported that the DPPH values for six different bread wheat grains ranged from 6.48 ± 1.46 µmol TE/g to 8.57 ± 1.46 µmol TE/g, which were significantly higher than our results. The genetic and environmental factors, the processing conditions [24,25], as well as the extraction procedures may all affect the antioxidant levels of wheat flour.

The DPPH values for bread made from whole wheat flour ranged from 2.79 to 4.05 µmol TE/g (mean 3.36 µmol TE/g), while bread made from refined flour ranged from 2.83 to 3.90, with mean 3.07 µmol TE/g. Again, bread made from refined flour had slightly higher values, except for “Great value”, which had a significantly higher value.

During the bread making process, the average scavenging ability decreased by approximately 32% and 30%, respectively, for whole wheat flour and refined flour. As explained above, the loss of scavenging ability could be attributed to the loss of some phenolic compounds, because the high temperature used during baking is known to cause the destruction of some phenolic acids [10]. Therefore, the extent of the decrease in DPPH scavenging abilities differed due to the different raw flour materials with varying phenolic contents.

### 3.3. Oxygen Radical Absorbance Capacity of Soluble Phenolic Compounds

Similar to the DPPH free radical scavenging activity, the ORAC values were expressed as micromole trolox equivalent per gram (µmol TE/g) of dry samples (Figure 1c). The refined flours (range 10.88 to 14.38 µmol TE/g, mean 12.52 µmol TE/g) showed significantly lower values compared to their whole wheat flour counterparts (range 27.93 to 44.33 µmol TE/g, mean 35.74 µmol TE/g) from the same brand.

The ORAC values of six varieties of wheat (hard/soft red spring, soft white/red winter, white spring durum and semi-hard red winter whole wheat grain) ranged from 19.58 to 37.49 µmol TE/g dry weight [1]. Another study investigated the antioxidant capacity of Canada Western Red Spring (CWRS). The ORAC value of whole grain was 95 ± 5 µmol TE/g, whilst that of white flour was 54 ± 2 µmol TE/g defatted material [26]. Compared with this literature, our results were in agreement with the previous study [1], but lower than those reported by the latter study [26]. Again, the genetic and environmental factors would affect the antioxidant levels of wheat [24].

ORAC values ranged from 51.89 to 64.65 µmol TE/g (mean 58.20) and from 24.93 to 44.16 µmol TE/g (mean 32.31) for whole wheat bread and refined bread, respectively. The bread samples demonstrated significantly higher ORAC values, which were 1.8 times and 2.9 times higher than their corresponding flour counterparts. The ORAC method is used to evaluate the ability of an antioxidant to scavenge the peroxyl free radicals [27]; therefore, phenolic compounds are more likely to be detected by this method through the donation of electrons. This also explains the reason for the increase in antioxidant activity after the bread making process, which was only detected by ORAC assay. Gelinas and McKinnon [11] also observed that the crust of white bread contained more phenolic compounds than the crumb, because of the Maillard reaction, and this phenomenon was better illustrated in white bread than wholemeal bread. 

According to Lin *et al*. [28], the improvement in antioxidant activity of wheat bread might be due to the incorporation of phenolic compounds, mainly rutin and quercetin; however, there was no further explanation of the incorporation. As Gelinas and McKinnon [11] indicated, Maillard reaction products (MRP) resulting from the Maillard reaction, a well-known non-enzymatic browning reaction, exhibited *in vitro* antioxidant activities. The Maillard reaction involves the interaction between amino acids and reducing sugars or lipid oxidation products [29]. The furan ring and nitrogen containing brown compounds (melanoidins) contributed to the antioxidant properties of baked grain products [30]. Water-soluble MRP, especially melanoidins, have been reported to have antioxidant activity according to the recently developed oxygen radical scavenging or chelating metal assays [29]. MRP could be another reason for the increase in antioxidant activity after baking, as detected only through the ORAC method. Moreover, Yilmaz and Toledo [29] also indicated that various factors, such as the type of reactants, temperature, pH, water activity and the availability of oxygen, could strongly influence the antioxidant properties of the MRP.

Michalska *et al*. [31] reported the antioxidant activity of rye bread using the ORAC assay. For whole bread, the ORAC value was 23.76 ± 1.64 μmol TE/g, while for white bread, the value was 14.4 ± 0.28 μmol TE/g dry material. In addition, Moore and Yu [32] reported the results on ORAC values for whole wheat bread as 20 µmoles TE/g and for white wheat bread as 12 µmoles TE/g. Compared with these values, our results were higher. Differences in the raw materials and bread making procedures could contribute to the discrepancies observed. The bread made by Michalska group was based on a longer fermentation time (totally 52 h) and baked at higher temperature for a longer time (260 °C for 40 min). The long fermentation time might lead to the destruction of antioxidants through decomposition by microorganisms or exposure to the air. The high temperature and longer time also affect the antioxidant activity.

### 3.4. Ferulic Acid Determination and Quantification Using HPLC

In wheat flour samples, ferulic, p-coumaric and vanillic acids were found to be the major phenolic acids in free forms [1], while syringic and protocatechuic acids were present in small amounts [33]. However, by comparing the retention time with 11 phenolic standards, even ferulic acid could not be detected when acidified aqueous ethanol was used as the solvent (results not shown). This would be attributed to the fact that ethanol extract may have only free ferulic acid, which contributes 0.1% of the total ferulic acid content. As previously reported by Adom and Liu [5], the free, conjugated and bound ferulic acids are in the ratio of 0.1:1:100. Therefore, alkali hydrolysis was used to release bound phenolics. 

**Figure 2:**
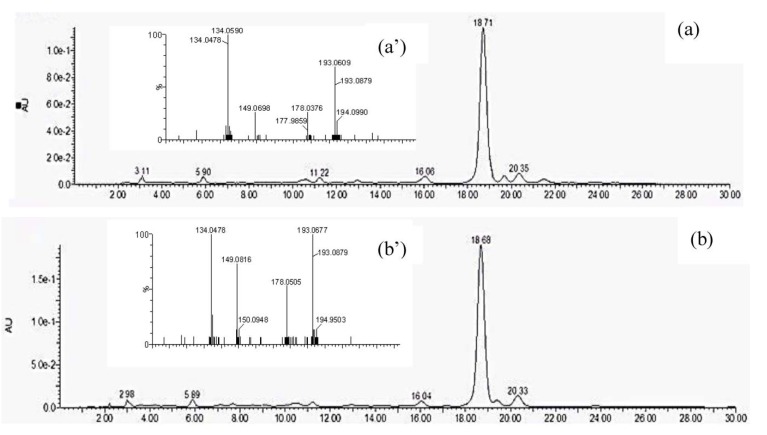
HPLC chromatograms of extracts of bound ferulic acid in the (**a**) flour sample and (**b**) bread sample and LC-MS/MS spectra of the (**a’**) flour sample and (**b’**) bread sample.

Figure 2 shows the HPLC chromatograms of bound phenolic acids from “Great Value” whole wheat flour and its corresponding bread. According to the chromatograms, ferulic acid (FA) was the dominant acid with a retention time of 18.7 min. Other peaks were too low and considered insignificant to demonstrate the effect of baking on flours. FA was the major phenolic acid in wheat grains (up to 85%–90%) [34] and present mostly in the bound form [35,36,37]. Thus, only ferulic acid (FA) was further quantified with HPLC. To confirm the identity, the LC-MS/MS spectra of the ferulic acid peaks in flour and bread were examined (Figure 2a’,b’). Based on the molecular ions [M − H]^−^ and fragmentation patterns, ferulic acid (*m/z* 193) provided a [M – H − 15]^−^ anion radical at *m/z* 178, due to the loss of the CH_3_ group (15 Da). Other fragments were generated at *m/z* 149 and 134, due to the loss of CO_2_ (44 Da) and CH_3_/CO_2_ groups. These assignments agreed with the mass spectra reported earlier [16,38].

The FA contents of flour and bread products are shown in Table 1. Bound FA levels in whole wheat flours (mean 271.6 μg/g) were significantly higher than in refined flours (mean 16.4 μg/g), because whole wheat flours contain both bran and germ fractions. Previous studies also have shown that the wheat phenolics were mainly concentrated in the bran and germ fractions of wheat kernels [14,39]. Hung *et al*. [40] reported that the content of bound ferulic acid in two Canadian wheat classes (whole wheat) ranged from 368 to 605 μg/g of the sample, values higher than those found in the current commercial flours (248 to 313 μg/g sample). The difference in endogenous levels of bound ferulic acid between the commercial flours and the two Canadian wheat classes could be due to several factors, including variety, environmental conditions during field production and post-harvest handling.

**Table 1:** Bound ferulic acid content (micrograms of ferulic acid (FA)/g of dry sample) in flour and bread products.

Sample Name	Whole Wheat Flour	Refined Flour	Bread Made from Whole Wheat Flour	Bread Made from Refined Flour
Robin Hood	247.8 ^ef^	14.5 ^g^	316.2 ^cd^	20.0 ^g^
Rogers	275.7 ^def^	22.7 ^g^	374.6 ^ab^	46.2 ^g^
No Name	290.2 ^cde^	20.8 ^g^	393.5 ^a^	29.2 ^g^
Great Value	313.1 ^cd^	11.5 ^g^	334.7 ^bc^	17.7 ^g^
Compliments	230.5 ^f^	12.8 ^g^	231.4 ^f^	13.6 ^g^

Different letters in each bar are significantly different at a level of *p* < 0.05 (Scheffe’s test).

The contents of bound ferulic acid were significantly higher in whole wheat bread compared to whole wheat flours, except for “Great Value” and “Compliments”. The increase was about 23% on average. The baking process did not significantly affect the endogenous low levels of ferulic acid in refined flour. Our results were in agreement with the studies of Gelinas and McKinnon [11] and Han and Koh [10], which indicated that the antioxidant activities of phenolic acids slightly decreased during mixing, but increased during fermentation and the baking process. Moreover, the HPLC analysis also explained the observations made using the ORAC method, which showed a higher antioxidant activity of bread samples.

## 4. Conclusions

Whole wheat flours demonstrated higher antioxidant activities than refined flours. They exhibited higher TPC, DPPH, ORAC and ferulic acid levels despite the presence of several additives in refined flour that partially compensate for the loss of germ and bran. The baking process reduced the TPC and DPPH scavenging capacity, but increased the ORAC values and ferulic acid content of bread. It appears that the bread making process released ferulic acid and enhanced compounds that scavenge the peroxyl free radicals, while destroying some of the reducing compounds that react with the Folin–Ciocalteu reagent and DPPH free radicals. The findings are in support of the body of literature that whole wheat products are likely to be more beneficial to consumers than the refined wheat products.
